# Acute Intermittent Porphyria: An Overview of Therapy Developments and Future Perspectives Focusing on Stabilisation of HMBS and Proteostasis Regulators

**DOI:** 10.3390/ijms22020675

**Published:** 2021-01-12

**Authors:** Helene J. Bustad, Juha P. Kallio, Marta Vorland, Valeria Fiorentino, Sverre Sandberg, Caroline Schmitt, Aasne K. Aarsand, Aurora Martinez

**Affiliations:** 1 Department of Biomedicine, University of Bergen, 5020 Bergen, Norway; helene.bustad.johannessen@uib.no (H.J.B.); juha.kallio@uib.no (J.P.K.); 2 Norwegian Porphyria Centre (NAPOS), Department for Medical Biochemistry and Pharmacology, Haukeland University Hospital, 5021 Bergen, Norway; marta.vorland@helse-bergen.no (M.V.); sverre.sandberg@noklus.no (S.S.); 3 INSERM U1149, Center for Research on Inflammation (CRI), Université de Paris, 75018 Paris, France; valeria.fiorentino@inserm.fr (V.F.); caroline.schmitt@aphp.fr (C.S.); 4 Norwegian Organization for Quality Improvement of Laboratory Examinations (Noklus), Haraldsplass Deaconess Hospital, 5009 Bergen, Norway; 5 Assistance Publique Hôpitaux de Paris (AP-HP), Centre Français des Porphyries, Hôpital Louis Mourier, 92700 Colombes, France

**Keywords:** acute intermittent porphyria, haem, hydroxymethylbilane synthase, enzyme intermediates, pyrrole chain elongation, porphobilinogen deaminase, pharmacological chaperones, protein stabilisation, proteostasis regulators

## Abstract

Acute intermittent porphyria (AIP) is an autosomal dominant inherited disease with low clinical penetrance, caused by mutations in the hydroxymethylbilane synthase (*HMBS*) gene, which encodes the third enzyme in the haem biosynthesis pathway. In susceptible *HMBS* mutation carriers, triggering factors such as hormonal changes and commonly used drugs induce an overproduction and accumulation of toxic haem precursors in the liver. Clinically, this presents as acute attacks characterised by severe abdominal pain and a wide array of neurological and psychiatric symptoms, and, in the long-term setting, the development of primary liver cancer, hypertension and kidney failure. Treatment options are few, and therapies preventing the development of symptomatic disease and long-term complications are non-existent. Here, we provide an overview of the disorder and treatments already in use in clinical practice, in addition to other therapies under development or in the pipeline. We also introduce the pathomechanistic effects of *HMBS* mutations, and present and discuss emerging therapeutic options based on HMBS stabilisation and the regulation of proteostasis. These are novel mechanistic therapeutic approaches with the potential of prophylactic correction of the disease by totally or partially recovering the enzyme functionality. The present scenario appears promising for upcoming patient-tailored interventions in AIP.

## 1. Introduction

**Figure 1:**
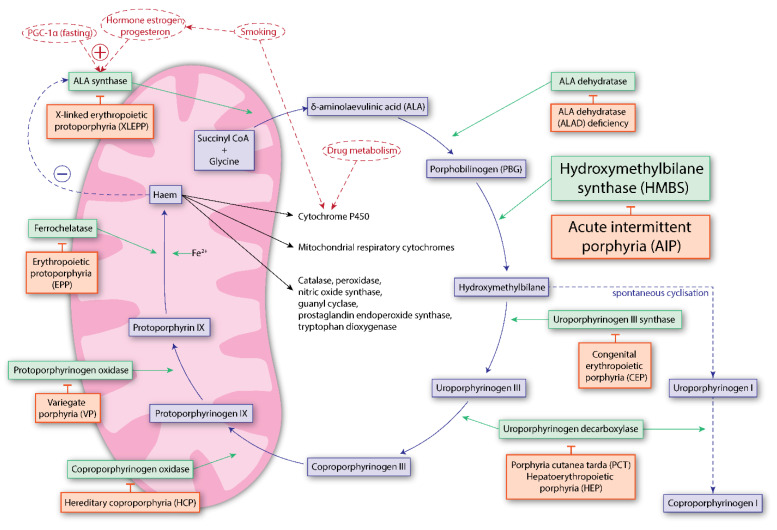
The haem biosynthetic pathway and associated porphyria disorders. In mammals, haem is synthesised in all nucleated cells through eight enzymatic steps and is essential for life. Each of the porphyrias (orange boxes) is caused by partial deficiency of the associated haem biosynthesis enzyme (green boxes), except for X-linked erythropoietic protoporphyria (XLEPP), which is caused by gain-of-function mutations of the erythroid-specific *δ-aminolaevulinic acid synthase 2* (*ALAS2*) gene. ALAS1, the housekeeping enzyme, is expressed in all other tissues. ALAS1 is under negative feedback control by haem (dashed blue line, left) and is rate-limiting under normal circumstances, when the catalytic capacities of the other enzymes in the pathway are normal. Drugs and hormonal factors have been identified as the most common precipitating factors for an acute attack (dashed red lines and sites of impact) [6]. Non-enzymatic and spontaneous cyclisation of hydroxymethylbilane to uroporphyrinogen I is an alternative path in cases of deficient uroporphyrinogen III synthase activity (dashed blue line, right). Figure modified from [7,8].

**Figure 2:**
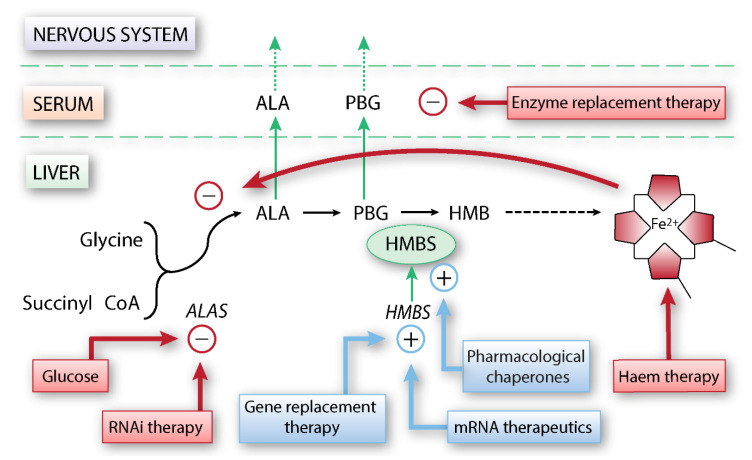
Overview of the action sites of established and potential therapy options for AIP. With a defect HMBS enzyme, ALA and PBG, produced in excess by the liver, leak into the bloodstream, and subsequently the nervous system. Therapeutic options that inhibit the pathway and/or accumulation of ALA and PBG are indicated in red, whereas options that enhance HMBS activity are shown in blue. Glucose treatment and RNAi therapy affect the *ALAS1* gene expression, ultimately downregulating the haem biosynthesis. Haem infusions inhibit the haem biosynthesis by negative feedback on ALAS1. Gene replacement therapy and mRNA therapeutics aid in delivering normal HMBS capacity and pharmacological chaperones aid in the expression of the HMBS protein or help the already-expressed HMBS protein to function correctly. Enzyme replacement therapy aims to supply functional HMBS to deplete surplus amounts of ALA and PBG. Figure modified from [9].

The porphyrias are a heterogeneous group of rare, inherited inborn errors of metabolism diseases, where each porphyria, except X-linked erythropoietic protoporphyria, results from a partial deficiency in one of the eight enzymes of the haem biosynthetic pathway [1]. The porphyrias can be classified as acute and/or cutaneous, depending on their clinical presentation (Figure 1). The most prevalent acute porphyria is acute intermittent porphyria (AIP), an autosomal dominant disease with low clinical penetrance, caused by deficiency in the third enzyme of the haem biosynthesis, hydroxymethylbilane synthase (HMBS). AIP is characterised by the overproduction of toxic haem precursors in the liver, resulting in so-called acute attacks, presenting with severe abdominal pain and a wide array of neurological and psychiatric symptoms. AIP is also associated with long-term complications in the form of primary liver cancer, hypertension, and kidney failure [2,3,4]. There are few established treatment options, with a complete lack of therapies that prevent the development of symptomatic disease and long-term complications in susceptible *HMBS* mutation carriers. Thus, patients with AIP and genetically predisposed individuals must adhere to lifelong lifestyle measures to reduce their risk of symptomatic disease [5]. From a mechanistic point of view, there are, however, many different potential treatment options for AIP. In this review, we provide an overview of established approaches, the status of relevant therapy, primarily gene-related, developments, and emerging therapeutic options, focusing on HMBS stabilisation and the regulation of proteostasis (Figure 2). We will also discuss the current structural information on wild-type HMBS and disease-associated variants, partly aiding in envisioning the complex kinetics of this enzyme, as well as information gaps that hinder a complete understanding of the oligopyrrole elongation mechanism.

### 1.1. Acute Intermittent Porphyria

AIP (MIM 176000) is caused by mutations in the *HMBS* gene [10]. More than 500 different disease-associated variants have been reported, with most being restricted to a few families. The disease is associated with a reduction in total HMBS enzyme activity by up to 50%, where the remaining activity derives from the presence of a normal allele expressing wild-type protein and any residual activity from the affected enzyme [11].

Although an autosomal dominant disease, AIP has low clinical penetrance. Studies examining genomic/exomic data reported a high prevalence of likely pathogenic *HMBS* variants (1 in ~1700), and thus a clinical penetrance of only 0.5–1% in the general Caucasian population [12,13,14]. However, in families with known AIP, penetrance has been estimated to be about 23% [13]. This underlines the importance of modifying environmental and/or other genetic factors in individuals with *HMBS* variants. In the European population, the incidence of symptomatic AIP has been estimated to 0.13:1,000,000 per year [15]. Predictive genetic testing of healthy at-risk relatives is recommended so that predisposed individuals can be counselled to reduce their risk of developing symptomatic disease [5]. *HMBS* mutation carriers who, during their lifetime, never experience symptoms of AIP may still present with highly elevated levels of δ-aminolaevulinic acid (ALA) and porphobilinogen (PBG), indicating metabolically active but asymptomatic disease [16,17]. Homozygous or compound heterozygous AIP is extremely rare and is associated with residual HMBS activity of less than 10% and chronic, neurodegenerative disease [18,19].

Symptomatic AIP is very rare in childhood [20], and the disease usually manifests after puberty, between the age of 15 and 40 years [21,22]. Acute attacks are more common in women than in men and are most frequently triggered by hormonal factors, such as those in relation to the menstrual cycle, or drugs [6]. Low calorie intake, physical/mental stress and infections are also reported as triggering factors [23]. Severe abdominal pain is the most typical symptom, usually accompanied by nausea and vomiting, intestinal motility disorders, muscle pain and tachycardia, in addition to electrolyte disturbances and a variety of neurological and psychiatric symptoms that may also be present [23,24]. Feared complications are severe hyponatremia and respiratory paralysis [25,26,27]. The biochemical hallmark of an acute attack is an increase in the haem precursors ALA and PBG, typically 10–20-fold the upper reference limit in urine. The other two autosomal dominant acute porphyrias, variegate porphyria and hereditary coproporphyria, which may also clinically present with bullous photocutaneous lesions due to porphyrin accumulation, are also characterised by increased ALA and PBG when in acute attacks. However, these are differentiated from AIP by the demonstration of a positive plasma fluorescence scan above 623 nm in the case of variegate porphyria and an increased faecal coproporphyrin III:I isomer ratio in hereditary coproporphyria [28]. Most AIP patients have increased ALA and PBG also in remission, often for years after an acute attack [29], which adds to the challenge of clinical evaluation of patients with AIP [30]. A small subgroup of patients, about 5%, experience recurrent acute attacks, usually defined as >4 acute attacks per year [15]. Recurrent patients, many of whom are younger women, typically have a high disease burden and diminished quality of life [31,32]. It has been shown that the tolerance for triggering factors differs even among members of the same family with the same mutation [33]. This indicates that the phenotypic outcome may be affected by other genetic and non-genetic factors [17] that influence the folding machinery’s efficacy or intracellular levels of substrate, which then affect the residual activity or stability of the mutant enzyme. There are, indeed, several reports describing a variation in the penetrance of selected mutations [13,17,34,35,36,37]. This source of phenotypic variability is probably the reason it has been difficult to establish clear genotype-phenotype correlations in AIP.

Long-term complications of AIP include hypertension, kidney failure and primary liver cancer [2,4]. Both symptomatic and asymptomatic *HMBS* mutation carriers have a highly increased risk of primary liver cancer compared to the general population [3,38,39,40] and are recommended annual/biannual liver surveillance, such as liver ultrasonography, after the age of 50 [3,41].

### 1.2. Pathophysiology of Acute Intermittent Porphyria

The haem biosynthesis pathway starts in the mitochondria with the condensation of glycine and succinyl CoA to ALA by ALA synthase (ALAS), followed by four enzymatic steps in the cytoplasm, and then back into the mitochondria, with three final steps resulting in the haem molecule (Figure 1) [1]. ALAS is rate-limiting if the catalytic capacities of the other enzymes in the pathway are normal. Two isoforms of ALAS are known: ALAS1 and ALAS2. ALAS2 is only expressed in erythroid cells whereas the housekeeping isoform, ALAS1, is expressed in all tissues and is under negative feedback control by haem. In the liver, drugs and chemicals that induce microsomal cytochrome P450-dependent oxidases (CYPs) induce ALAS1 activity. For a patient with AIP, in situations with increased demand for haem, a deficient HMBS enzyme becomes rate-limiting in the haem biosynthesis in the liver. The increased flux through this step causes accumulation of the haem precursors such as ALA and PBG [42]. ALA is most likely the metabolite causing the neuropathic symptoms in AIP [43,44,45]. Actually, in vitro studies established ALA as a neurotoxin [46], and oxidation of ALA resulting in reactive oxygen species (ROS) [47] was early proposed to be related to neuropathy in the acute attacks [48,49]. The toxic effects of ALA have been reported to be concentration-dependent and associated with ROS [50]. DNA damage because of ALA-induced ROS has also been implicated in the development of primary liver cancer in AIP [51]. Through the work with ALA-based photodynamic therapy, ALA has also been connected to pain, possibly by tissue damage by ROS and nerve stimulation, via its resemblance to γ-aminobutyric acid (GABA) and as a substrate of GABA transporters (GATs) [52,53]. ALA also interacts with GABA receptors [54] and inhibits cellular uptake of GABA [55]. Nevertheless, the complete picture is not yet understood, particularly considering *HMBS* mutation carriers who have high concentrations of ALA without ever experiencing symptomatic disease [16,17]. Marked increases in urinary ALA concentrations are also observed in lead toxicity and ALA dehydratase (ALAD, also known as porphobilinogen synthase) deficiency, both affecting the second enzyme in the haem biosynthesis (Figure 1) [1]. In lead toxicity, zinc in the ALAD metal-binding site is displaced by lead [56], which inhibits ALAD catalytic activity and subsequently causes increased ALA excretion and, in some patients, symptoms mimicking the acute attacks seen in AIP [56]. Lead poisoning has also been described to be associated to changes in the quaternary structure of ALAD by shifting the oligomeric equilibrium toward the ligand-bound, low-activity hexamers, a mechanism which has also been suggested to explain the toxicity of certain drugs [57,58]. ALAD deficiency porphyria, which is an extremely rare autosomal recessive disorder, is characterised by a marked increase in urinary ALA, in combination with normal or only slightly increased urinary PBG concentration [59]. The clinical presentation resembles AIP, with patients suffering from acute neurovisceral attacks or chronic neuropathy [60]. Patients with hereditary tyrosinemia may also present with increased ALA excretion and experience neurologic crises comparable with those seen in AIP [61]. In this disorder, succinylacetone accumulates and, being structurally similar to ALA, causes inhibition of ALAD. In patients with AIP, family studies have shown that genetic and environmental factors affect the risk of developing symptomatic disease in *HMBS* mutation carriers [13]. In addition to factors influencing ALAS1 activity (see above), conditions affecting ALAD activity, e.g., by changing its quaternary structure [58], may be likely to also modulate ALA concentration. Thus, it is possible that ALAD modifications may contribute to the variation in ALA concentrations observed in *HMBS* mutation carriers.

### 1.3. Hydroxymethylbilane Synthase

**Figure 3:**
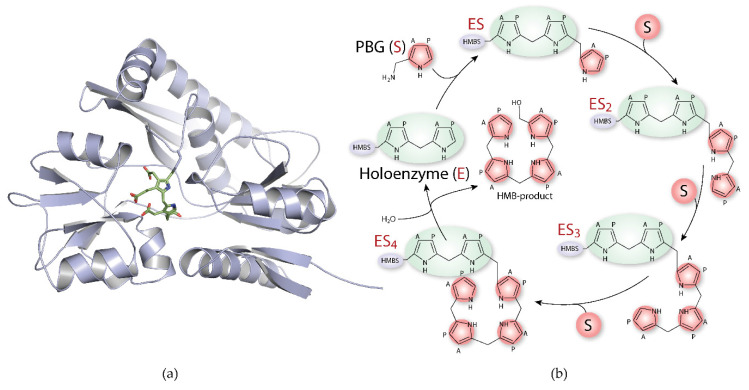
The pyrrole chain elongation catalysed by hydroxymethylbilane synthase. Hydroxymethylbilane synthase (HMBS) and the pyrrole chain elongation. (**a**) Cartoon representation of HMBS crystal structure (blue; PDB ID: 3ECR) with the dipyrromethane (DPM) cofactor in the centre. The housekeeping HMBS is a monomeric protein of 39.3 KDa. (**b**) HMBS (blue) with the DPM cofactor (2 × PBG molecules; green) constitute the holoenzyme (E) and bind four PBG substrates (S; red), sequentially, producing enzyme intermediates ES (=ES_1_), ES_2_, ES_3_ and ES_4_. Hydroxymethylbilane (HMB), the linear tetrapyrrole product, is released by hydrolysis. The substrate sidechains are acetate (A) and propionate (P). Figure modified from [76,77].

HMBS (EC 2.5.1.61; also known as porphobilinogen deaminase, uroporphyrinogen I synthase) catalyses the assembly of four PBG molecules into the linear tetrapyrrole 1-hydroxymethylbilane (HMB; also known as preuroporphyrinogen), the precursor of uroporphyrinogen III. The pyrrole elongation (Figure 3) is a stepwise condensation, which starts from a dipyrromethane (DPM) cofactor in the holoenzyme (E), creating enzyme-intermediate complexes bound to one, two or three PBG molecules, referred to as ES, ES_2_ and ES_3_. In the last step, an enzyme-intermediate complex bound to four PBG molecules is generated, i.e., ES_4_, and quickly released from the cofactor by hydrolysis yielding the linear HMB, as reviewed by Bung et al. [62]. The DPM cofactor, composed of two PBG molecules, is not incorporated in the product and remains covalently attached to the enzyme [63,64].

Louie et al. solved the first crystal structure of the HMBS enzyme in 1992 (PDB ID: 1PDA [65]). Although the enzyme originated from *E. coli*, it shares a high degree of sequence identity with human HMBS and provided valuable information for the structure and function of the enzyme. Common structural features were confirmed by Song et al. in 2009, when the first crystal structure of human HMBS was published (3ECR [66]). Since then, multiple human HMBS crystal structures have been published, and these are summarised in Table 1, with key findings obtained from the structures. Despite the valuable information gained from these structural results, the details of the complete catalytic mechanism are yet to be solved.

**Table 1:** Summary of the published crystal structures of HMBS enzyme from various organisms. Presenting the key structural aspects and highlights from the publications.

PDB	Res. [Å]	Organism (UniProt)	State	Mutations	Areas NOT Defined in the Electron Density (Total Number of Residues)	Key Findings	Ref.
1PDA	1.76	*E. coli* (P06983)	E	—	A:1–2, 49–57, 308–313 (313)	First HMBS structure description. Already suggest movement in domain 3 during the elongation process. Importance of D84 (D99 in human HMBS). Sequence comparison to higher organisms.	[65]
1YPN	2.3	*E. coli* (P06983)	E	K59Q	A:1–2, 48–57, 307–313 (313)	K59Q affects the rate of intermediate formation. Crystal packing inhibits the domain movements in time-resolved study.	[67]
1AH5	2.4	*E. coli* (P06983)	E	Se-Met labelled	A: 1–2, 47–58 (313)	Seleno-methionine labelling for phasing. Structural study to confirm suitability to time-resolved experiments.	[68]
2YPN	2.3	*E. coli* (P06983)	E	—	A:1–2, 43–59 (313)	Methodology based research on Laue diffraction.	[69]
1GTK	1.66	*E. coli* (P06983)	E	—	A:1–2, 43–59 (313)	Highest resolution structure for *E. coli*	[70]
3ECR	2.18	*H. sapiens* (P08397)	E	—	A: 1–17, 57–75, 259–260, 357–361 (361) B: 1–18, 57–69, 259–260, 301–312, 356–361 (361)	First to describe human HMBS structure. Confirming similarity to *E. coli* HMBS. Mapping disease related mutants on the human HMBS structure. Suggesting a role in domain movements for H120.	[66]
3EQ1	2.8	*H. sapiens* (P08397)	E	R167Q	A: 1–18, 56–76, 303, 307–310, 357–361 (361) B: 1–18, 56–76, 303, 307–310, 357–361 (361)	Specific activity of R167Q HMBS is lowered to 10% compared to wild-type. Accumulation of intermediates detected using native PAGE.	[71]
4HTG	1.45	*A. thaliana* (Q43316)	E	—	A: 1–9, 312–320 (320) ^1^	Cofactor in oxidised form. First structured active-site loop. Highest resolution HMBS structure to date.	[72]
4MLQ 4MLV	1.6 1.45	*B. megaterium* (Q8GCA8)	E	—	A: 40–60, 309–310 (310) A: 40–60, 310 (310)	Mixture of oxidised and reduced form for the cofactor.	[73]
5H6O	2.7	*V. cholerae* (Q9KVM1)	E	—	A:1–3, 43–61, 304–311 (311)		—
5OV4 5OV5 5OV6	2.69 1.81 1.87	*B. megaterium* (Q8GCA8)	E E E	D82A D82E D82N	A: 40–60, 310 A: 41–60, 310 A: 40–59, 310 (310)	By mutating D82, binding/formation of the cofactor could be altered, and enzyme inactivated. D82 corresponds to D99 in humans.	[74]
5M6R 5M7F **^2^**	2.73 2.78	*H. sapiens* (P08397)	ES_2_ E	—	A: 1–17, 358–361B: 1–17, 62–75, 354–361 (361) A: 18–19, 58–74, 354–361 B: 18–19, 57–74, 354–361 (344)	First ES_2_ crystal structure. Partially ordered structure for the active-site loop.	[75]
7AAJ 7AAK	1.8 1.7	*H. sapiens* (P08397)	E ES_2_		A,B: 1–17, 62–75, 354–361 (361) A: 1–17, 358–361 B: 1–17, 62–75, 354–361 (361)	Highest resolution human HMBS structure. First disease mutant structure to show accumulation of one intermediate. Partially ordered structure for active-site loop.	[76]

^1^ Sequence of *A. thaliana* HMBS (UniProt ID Q43316) includes a 62-residue long transit peptide for chloroplast, with total length of the encoded protein 382 residues. ^2^ Expressed as erythroid specific isoform, which contains residues 18–361 of the full-length isoform. Abbreviations: E corresponds to enzyme with the dipyrromethane cofactor, and ES_2_ to the tetrapyrrole intermediate.

## 2. Current Treatment Options for Acute Intermittent Porphyria

### 2.1. Established Treatments for Sporadic and Recurrent Acute Attacks

Haem infusions, in the form of haemin (Panhematin^®^, Recordati Rare Diseases, Xellia Pharmaceuticals USA, LLC, Buffalo Grove, IL, USA) [78,79] or haem arginate (Normosang^®^, Recordati Rare Diseases, Puteaux, France) [80] are presently, in most settings, the preferred specific treatment for sporadic acute attacks [24,25]. Haem restores hepatic haem and consequently downregulates ALAS1 through the negative feedback loop in the haem biosynthesis (Figure 2) [81,82]. Haem infusions are usually well tolerated and successful in the treatment of sporadic acute attacks. However, when used as an off-label prophylactic treatment in patients with recurrent attacks, the maintenance of central venous access, development of iron overload and the possible decrease in efficacy during prolonged or repeated haem therapy are challenging [15,25,83,84]. Furthermore, studies indicate that frequent haem infusions may induce hepatic haem oxygenase 1, which causes increased ALAS1 activity, thus maintaining disease activity [85]. High-carbohydrate infusions are also used for treatment of sporadic attacks, either as a stand-alone treatment for a less severe attack, in combination with haem infusions or as the main treatment in countries where haem is not available [24]. The effect of this treatment is, however, disputed. It has been shown in mice that glucose suppresses the expression of peroxisome proliferator-activated receptor γ coactivator 1α (PGC-1α), a transcriptional coactivator factor important for controlling the expression of ALAS1 [86]. The subsequent increase in insulin activates the protein kinase AKT [87,88], also leading to the inhibition of PGC-1α. For women experiencing recurrent attacks related to the menstrual cycle, a gonadotropin-releasing hormone analogue can be given, but it has limited effect and, for many patients, unacceptable side effects [89,90].

### 2.2. Liver and Kidney Transplantation

For severely affected AIP individuals, typically patients with strong and recurrent acute attacks, liver transplantation has been used in the last decade as a last-resort treatment alternative [91]. In acute hepatic porphyrias, the liver is the main source of the overproduction of haem precursors, and liver transplantation corrects the metabolic defect, normalising ALA and PBG excretion [92,93]. Although most patients with liver-transplant gain a better quality of life, with a survival rate in line with other indications for liver transplantation, it is not an intervention without challenges or risk, and there is also a limitation in available donors [93,94,95].

Based on a large French AIP cohort, as many as 50% of AIP patients will experience porphyria-associated kidney disease, likely resulting from the effect of ALA on the tubular cells [4]. Variants in the peptide transporter 2 (PEPT2) have been shown to be associated with the severity of porphyria-associated kidney disease [96]. PEPT2 has a central role in ALA tubular reabsorption, and the use of inhibitors of this transporter, such as the angiotensin II receptor antagonist losartan, has thus been suggested as a potential nephroprotective strategy in AIP [96]. In some cases, AIP patients develop severe end-stage renal disease [97]. Kidney transplantation may, in these patients, be therapeutic, eliminating or reducing the frequency of AIP attacks and resolving skin lesions likely caused by spontaneously formed uroporphyrins, which are only poorly filtered across the dialysis membrane [98]. Combined liver and kidney transplantation have also been reported for severely affected AIP patients with renal failure, with successful outcome [99,100].

### 2.3. Ribonucleic acid (RNA) Interference Therapy

Givosiran (GIVLAARI^®^, Alnylam Pharmaceuticals, Cambridge, MA, USA) is a ribonucleic acid interference (RNAi) therapy using synthetic small interference RNA (siRNA) to reduce hepatic *ALAS1* messenger RNA (Figure 2) [101] conjugated to *N*-acetyl-galactosamine for liver-specific delivery [102]. Phenobarbital induces the overexpression of ALAS1 in *Hmbs*-deficient mice, which leads to the accumulation of ALA and PBG in both plasma and urine and, therefore, acute attacks [103]. The administration of siRNA to *Hmbs*-deficient mice during an ongoing acute attack reduced both ALAS1 expression and ALA/PBG levels, while prophylactic administration of siRNA prevented phenobarbital-induced attack as a proof of concept for siRNA as a mechanism to prevent and treat acute attacks [103].

Givosiran was approved for the treatment of adults with acute hepatic porphyria in November 2019 in the US and received a positive opinion in January 2020 in EU for treatment in adults and adolescents aged 12 years and older [104]. The approvals were based on the positive report of the phase III trial ENVISION [105]. ENVISION included recurrent acute hepatic porphyria patients, the overwhelming majority of whom had AIP, and major findings were a significant reduction in both attack rates and ALA and PBG concentrations in the givosiran group as compared to the placebo group. Reported key adverse events were related to the liver and kidney function, with increases seen in serum aminotransferase levels and the estimated glomerular filtration rate in givosiran-treated participants. Long-term effects and efficacy are still unclear, as only data for the first 6 months’ period of the phase 3 study have been published in detail [106]. Givosiran is now being introduced into clinical practice to treat severely affected patients with recurrent acute attacks. Based on reported side effects and observation of the treatment of patients, this siRNA therapy requires close follow-up of patients, with a particular focus on kidney function, liver enzymes, lipase and homocysteine.

## 3. Other Potential Therapeutic Developments

### 3.1. Enzyme Replacement Therapy

Development of enzyme replacement therapy (Figure 2) was attempted in the early years of 2000. Treatment with intravenously administered recombinant human HMBS demonstrated some biochemical evidence and potential in both mice [107] and symptom-free individuals by decreasing the plasma and urinary concentrations of ALA and PBG [108]. However, it proved to be unsuccessful in clinical trials [109], possibly due to the intravenous infusion of erythroid HMBS without being targeted to the liver [24] and has not been developed further.

### 3.2. Gene Replacement Therapy and mRNA Therapeutics

In gene replacement therapy, the correct *HMBS* DNA or mRNA sequence is transferred to liver cells to restore normal HMBS activity (Figure 2). Nonviral gene vectors aiming to correct AIP were first reported by Johansson et al. [107,110]. Proof of concept of gene therapy was shown in a study with transgene-expressing, first-generation recombinant adenovirus, where *Hmbs*-deficient mice were protected from attacks induced by phenobarbital [111]. However, the treatment did not show the long-term effects and could not be repeated due to immunisation [111]. Full and sustained protection from both the accumulation of ALA and PBG and acute attacks in *Hmbs*-deficient mice was obtained in subsequent investigations, using a helper-dependent adenovirus carrying the *HMBS* gene and a liver-specific promoter [112]. This vector was, however, not suitable in clinical trials. An adeno-associated virus (AAV) vector, devoid of the genes necessary for viral gene expression, was also explored, and two independent research groups demonstrated non-toxic and long-term effects in the *Hmbs*-deficient mouse model [113,114]. The AAV2/5-HMBS orphan drug was proven to be safe and with therapeutic effects in cynomolgus monkeys [115]. The phase I clinical trial showed that administration of the recombinant AAV gene vector was safe, but without metabolic correction [116,117]; it was suggested that the vector doses were unable to induce sufficient liver transduction. This issue was addressed by the construction of an improved gene therapy vector with an inducible promotor responsive to factors like oestrogens, starvation and certain porphyrinogenic drugs and by the construction of a hyperfunctional bioengineered *HMBS* variant, with both approaches showing promising results in *Hmbs*-deficient mice [118,119].

Promising results have also been obtained in a preclinical study using intravenous human *HMBS* mRNA encapsulated in lipid nanoparticles (Moderna Inc., Cambridge, MA, USA). This study showed an abundant HMBS protein expression in hepatocytes of *Hmbs*-deficient mice and rapidly normalising urine porphyrin precursor excretion in ongoing attacks, and in a chemically induced acute porphyria rabbit model, thus providing proof-of-concept for systemic human HMBS mRNA as a potential therapy for AIP [120]. Repeat dosing in *Hmbs*-deficient mice showed sustained efficacy and therapeutic improvement without evidence of hepatotoxicity, and translatability was demonstrated by multiple administrations to nonhuman primates [120].

### 3.3. Hepatocyte Transplantation

Hepatocyte transplantation is less invasive and less expensive than liver transplantation, with fewer side effects and with the possibility of repeating the engraftment procedure [121], thus offering numerous advantages compared to traditional transplantation approaches. The transplantation of wild-type hepatocytes into the liver of *Hmbs*-deficient mice indicated an approximately 50% reduction in ALA and PBG in plasma in transplanted mice compared to non-treated mice after phenobarbital induction [122]. In addition, already-accumulated ALA and PBG was metabolised by transplanted hepatocytes, with both results suggesting that a correction in a fraction of the cells may be enough to improve clinical AIP symptoms. However, further follow-up studies on hepatocyte transplantation for correction of AIP have not been published, and long-term improvement in metabolic deficiencies is an issue in need of further investigation [123].

## 4. Protein Stabilisation and Proteostasis Regulation—Emerging Treatment Opportunities

### 4.1. Protein Folding and Stability in HMBS

For most proteins, a defined three-dimensional conformation with inherent stability and flexibility is needed to maintain their specific function and regulation. The folding into the functional native state conformation is driven by free energy (*G*), often via folding intermediates, aiming towards the lowest energy folded state, as classically presented by the funnel-shaped energy landscape model [124,125]. The complete network of weak non-covalent interactions between all residues determines the stability of the resulting protein structure, where the cumulative contributions add up to considerable favourable energetics. The view of the native state is, however, expanding from being a unique conformation with the lowest-energy three-dimensional structure, to comprise fluctuating conformational ensembles. The inherent dynamics of any protein are chemically coded in the protein sequence and structure and are linked to its function [126], allowing it to adapt and alternate its conformation as a response to environmental stimuli and ligand binding at different times or locations [127]. Furthermore, the characterization of intrinsically disordered proteins, metamorphic proteins, chameleonic sequences and morpheeins [128,129,130,131] shows that the classical funnel-shaped energy landscape has clear shortcomings, as these folding arrangements imply low energy barriers between states to alternate in fine-tuned shifting equilibria [127,132]. Thus, as the need for flexibility and dynamics associated with proper function usually decreases protein stability, native state ensembles appear to balance fluctuations, function and stability, in a quest where electrostatic surface interactions appear important to modulating folding barriers in response to special functional requirements within a given structural fold [126].

Wild-type HMBS is a monomeric protein and considered to be very stable based on thermal denaturation and thermal-dependent functional studies, with a half-denaturing temperature (*T*_m_) of ~75 °C [35,37,133], whereas a large number of disease-related variants of HMBS that have been studied as isolated proteins present with reduced thermal stability [12,35,37,134,135,136]. Interestingly, thermostability assays of isolated enzyme intermediates (E_(holo)_ and ES_1–4_; Figure 3) have revealed that the enzyme loses thermal stability upon pyrrole chain elongation, most probably associated with the increased mobility and enlarged active site required to accommodate the growing product [37]. The instability associated with catalysis may explain the increased in vivo degradation of intrinsically active but unstable HMBS mutants [137]. However, a more interesting consequence of this observation is that, as the native state ensemble in HMBS equilibrates a variety of metastable alternate conformations representing each intermediate elongation state may occur [138], and it might be inferred that some AIP-associated variants may alter the populations of these intermediates. This indicates that in addition to the classical view, where mutations often cause a decrease in the conformational stability of the native-like state, for some mutations the altered distribution of the intermediates may lead to the observed loss of thermal stability and decreased HMBS activity, opening up an option for strategies to remodulate the distribution of the populations by specific ligand binding (see also Section 4.4).

**Figure 4:**
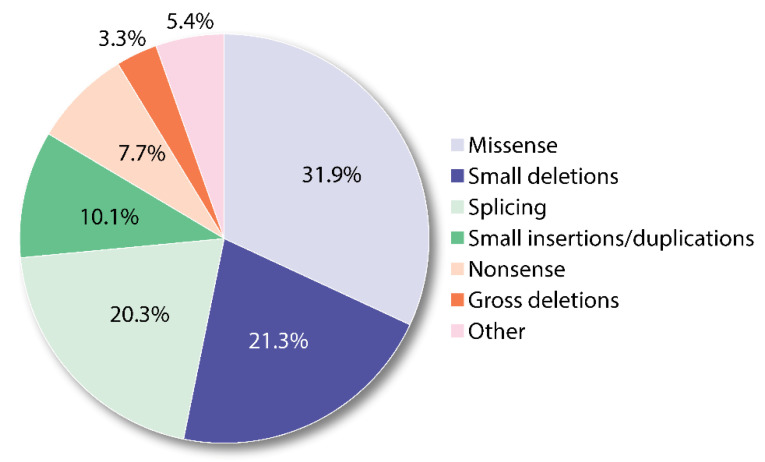
Overview of reported *HMBS* variants. The numbers are based on 517 variants included in the 2020.3 Human Gene Mutation Database (HGMD^®^; www.hgmd.cf.ac.uk/ac/gene.php?gene=HMBS) and the variants are grouped into different mutation types. The category ‘Other’ includes small indels, 2.7%; regulatory, 1.4%; gross insertions/duplications, 0.8%; complex rearrangements, 0.6%.

Of the more than 500 reported AIP-associated HMBS variants, the majority are missense (31.9%) followed by small deletions, insertions and duplications (Figure 4). Supplementary Table S1 provides an overview of the classification of missense variants, the current status of functional and biochemical studies, and original conclusions on the functional effect [8,12,13,37,62,134,135,136,139,140,141,142,143,144,145,146,147,148,149]. The majority of the missense mutations have been associated with a catalytic deficiency, where, in particular, p.Arg116Trp presents a severe conformational defect and is reported as unstable in several studies (Supplementary Table S1 and [37]). On the other hand, several other mutations indeed show an altered distribution of enzyme intermediates, where p.Arg173Trp is a clear example, as only the ES_2_ intermediate state is observed by native PAGE [37]. Recent high-resolution mass spectrometry and X-ray crystallography studies have indeed demonstrated how this mutation may alter the function of HMBS, which is trapped in a non-functional ES_2_ intermediate state [76] (see also Section 5).

### 4.2. Proteostasis Regulation

A current view of inborn errors of metabolism is that a majority of them are loss-of-function conformational disorders, where the decreased activity—in the case of enzymes—is explained by a direct effect of the mutation on the catalytic efficiency (changes in *K*_cat_, *K*_M_ or both), or by increased protein instability and thus premature degradation. Protein homeostasis, or proteostasis, refers to cellular processes that control the biosynthesis, folding, trafficking and degradation of proteins [150,151]. Whereas mutation-associated instability often results in accelerated protein degradation, resulting in loss-of-function, mutations may also lead to non-functional misfolding, oligomerisation and aggregation that can compromise proteostasis and cause a cytotoxic gain-of-function [124,152]. Newly translated nascent proteins often require assistance from molecular chaperones to acquire their correct native conformation and functional distribution of conformers in the protein ensemble, as well as to avoid an aggregation of non-native conformations [138,153,154]. Essential strategies of the proteostasis network for proteins that are recognised as incorrectly folded and/or unstable are refolding, degradation, or spatial sequestering, mainly via inspection and maintenance by molecular chaperones, the ubiquitin–proteasome system (UPS) and autophagy [124,155]. Proteins that cannot be refolded will be labelled with one or several covalently attached ubiquitin units, becoming recognisable to the UPS and the autophagy system, and consequently degraded [124,153]. There is an efficient crosstalk between the UPS and the autophagy system, and aggregated proteins that cannot be processed by the UPS are either engulfed and degraded directly in lysosomal invaginations, or via autophagosomes by selective autophagy receptors such as p62/SQSTM1 [156].

Protein aggregation is a hallmark in conformational neurodegenerative disorders, where the accumulation of aggregates appearing as soluble oligomers and/or amyloid fibrils causes toxic gain-of-function [157,158], as these aggregates often overwhelm the autophagy system, causing cellular toxicity [124,159]. Protein aggregation is also observed in genetic metabolic disorders, such as monogenic forms of diabetes [160]. There is also an increasing awareness of the pathogenic seeding effect of amyloid-like assemblies of metabolites that accumulate in inborn error of metabolism, promoting protein aggregation [161]. There is, to our knowledge, no report on the formation of aggregates of HMBS variants in liver or other organs in AIP. However, for cutaneous and photosensitive porphyrias, it has been shown that fluorescent porphyrins protoporphyrin-IX, uroporphyrin and coproporphyrin, which are photoreactive, cause oxidative stress and can result in the formation of protein aggregates and tissue damage [162]. Oxidative stress appears to be a recurrent finding in AIP [163] and we cannot disregard the possibility that severe proteostasis dysregulation, even leading to certain gain-of-function aggregation, occurs in AIP.

### 4.3. Therapeutic Strategies Based on the Regulation of the Proteostasis Network

Large-scale genomics analysis in models of protein conformational disorders (a term that includes disorders associated with both loss-of-function and gain-of-function instability) has revealed that natural genetic variation may have an important modulatory effect on the phenotype of the disease [164]. Genetic variation in components of the proteostasis network may thus explain the variations in the penetrance and severity of the disease, and the noticeable deviations of the genotype–phenotype correlations that characterise a complex trait disorder, such as Fabry disease [165], an attribute that recently has also been associated with AIP [13,139]. This understanding suggests that strategies to restore proteostasis, by either enhancing the folding capacity of the proteostasis network, or modulating the degradation of the incorrectly folded proteins, may represent novel therapeutic strategies for conformational diseases [166]. Target-specific pharmacological chaperones are alternative possibilities (see below) and these approaches have often been applied in combination [165].

The use of either small molecules (e.g., celastrol) or biologics (e.g., siRNA) that enhance the capacity of the proteostasis network in the relevant cellular compartments, has shown promising results in the case of lysosomal storage disorders, such as Gaucher’s disease, as well as for cystic fibrosis caused by ΔF508 CFTR [165,167]. To our knowledge, studies with these types of proteostatic regulators have not been reported for AIP. In other metabolic disorders, strategies to interfere in the degradation by the UPS have been shown to counteract the premature breakdown of unstable mutants [168]. Possibilities for interference targets include molecular chaperones and deubiquitinating enzymes, as well as UPS inhibitors. The clinically approved proteasome inhibitor bortezomib has been shown to be effective in reducing porphyrin accumulation as well as in reversion of the skin photosensitivity of the mouse models of congenital erythropoietic porphyria, which is associated with mutations in the uroporphyrinogen III synthase gene (Figure 1) [169,170]. One of the pitfalls of the use of proteasome inhibitors is, however, that they may negatively affect many other cellular processes. The use of deubiquitinating enzymes, combined with technologies that infer specificity, may provide a more regulated stabilisation of mutated targets [171].

### 4.4. Pharmacological Chaperones

Pharmacological chaperones are small-molecule compounds that bind selectively to protein targets and stabilise the functional state and/or facilitate the folding of non-native intermediate states toward the native protein, enabling a partial or total recovery of the functional conformation [137,172,173] and/or a correct trafficking within the cell [174]. Thus, effective pharmacological chaperones prevent unstable, yet potentially active proteins from detection and degradation by the quality control system, and consequent reduction in the proteostasis network overload [175,176]. An example can be seen for the anti-prion cation tetrapyrrole Fe-TMPyP that, in addition to stabilising the native state of prion protein PrP, acts as a suppressor of non-native inter-domain interactions by binding to unfolded PrP and inhibiting the formation of stable aggregates [177]. Furthermore, Fe-TMPyp also binds to monomeric intrinsically disordered α-synuclein and inhibits formation of fibrils and small oligomers [178].

In the case of inborn errors of metabolism, the pharmacological chaperone therapeutic concept can be applied to, among others, prolonging the half-life of unstable mutants in vivo, to allosteric activate enzymes and to achieving functional cellular localization [179,180]. Supplementation with elevated concentrations of synthetic forms of natural ligands and cofactors has also been successfully used to protect the mutant proteins against accelerated degradation [181,182]. Nevertheless, the mechanism of action of pharmacological chaperones is not completely understood, and other mechanistic scenarios have been put forward [183,184]. Considering that mutations may change the distribution of the conformational ensemble, e.g., by enhancing the relative proportion of less active conformers with respect to the active conformation, specific ligand binding to the active conformers may shift the equilibrium towards the accumulation of these functional conformers in the mutant protein, increasing its activity [132,183,185]. Moreover, several binding mechanisms have been proposed, but, commonly, they describe a specific interaction of the pharmacological chaperones in an explicit location of the target protein. Two main binding modes have emerged: (i) the active-site-specific, related to the substrate, also called classical pharmacological chaperones, and (ii) non-active site—often called allosteric—pharmacological chaperones [180,186,187]. The active-site-specific pharmacological chaperones bind reversibly in the active site with sufficient binding affinity to increase the stability of the enzyme and can be replaced by the substrate in protocol balancing stabilisation (on mode) and catalytic function (off mode, e.g., by stopping the administration for increased activity) [188,189,190]. Counter-intuitively, these compounds may function as competitive inhibitors; however, the effects of preventing destabilisation and misfolding, as well as shifting the conformer equilibria towards an active “substrate- or cofactor-bound like state”, are expected to outbalance the inhibition [189]. The non-active site or non-inhibitory pharmacological chaperones interact with the target protein outside the active site and may also induce a subsequent allosteric motion [191,192]. Such chaperone compounds can be bound to the target proteins without inhibiting catalysis at the active site and may allosterically induce a more active conformation [193,194,195].

In the search for stabilising compounds to develop a pharmacological chaperone therapy, experimental medium-high-throughput screening of a large library is a common initial step; however, different initial assays can be applied to select effective hit compounds. One method is to screen for compounds affecting activity in cells stably expressing target-protein [196] or patient-derived mutant enzymes [197]. However, since active-site-interacting compounds with an activity-enhancing effect in vivo may exhibit mild inhibition in vitro [189] an activity-based assay may fail to identify these hits. Instead, screening for compounds that can bind and stabilise the recombinantly expressed protein, followed by counter-assays to validate binding, target-stabilisation and effect on activity can be applied [198,199,200]. Moreover, computational approaches such as the structure-based virtual screening of compound libraries by docking to the target protein structure aim to discriminate between binders and non-binders [201]. Ligand-based virtual screening approaches, where chemical features of known ligands are used to prepare a pharmacophore that is then targeted in ligand-based screening, can be used [202]. In both experimental and computational approaches, large libraries (up to 10^7^ molecules) can be screened, and hits derived from computational screenings are then validated experimentally.

In heterozygous AIP cases, representing most patients, fully functional wild-type HMBS is expressed from one allele [11]. In a recent study, we therefore targeted the wild-type enzyme in the search for non-inhibitory pharmacological chaperones, aiming to correct and treat AIP independently of the patients’ mutation. The proof-of-concept was demonstrated in *Hmbs*-deficient mice, where the excretion of urinary ALA and PBG decreased, and enzyme levels and activity of HMBS in liver lysates increased upon administration of the hit compounds 5-[(2-chlorophenyl)methyl]-2-hydroxy-3-nitrobenzaldehyde and 4-chloro-3-nitrophenyl(phenyl)methanone [133]. These compounds bind close to the HMBS active site, only slightly affecting the catalytic constants and decreasing the proteolytic degradation of the enzyme. Subsequent hit expansion and hit-to-lead optimization programs are expected to result in derivatives of these compounds with increased affinity and improved pharmacological properties, which can be developed into an oral AIP treatment that could work curatively during acute attacks, but also prophylactically.

Another possibility in the search for stabilising agents is the repurposing of already marketed, off-patented drugs, as has been demonstrated for congenital erythropoietic porphyria [203]. This approach may also be more cost-efficient than the more traditional search for novel medicines, as repurposed drugs are generally approved sooner, and their development costs are lower [204].

## 5. Structural and Mechanistic Challenges of HMBS for Therapeutic Developments

Despite recent development in the field of HMBS by the determination of the X-ray structures of the human wild-type holoenzyme and ES_2_ intermediate state [75,76], the details of the enzyme elongation mechanism and product release are still unclear (Figure 3). Knowing the detailed mechanism is crucial for the understanding of different clinical phenotypes in patients, and why some disease-related variants affect the enzyme activity without obvious indications of altering the holoenzyme structure. Furthermore, an understanding of the catalytic mechanism can also pave the way for novel therapeutic approaches.

X-ray crystallography is a routine technique to determine the atomic and molecular structure of a crystal. Regarding proteins, it can give detailed information on protein structure, function, and interactions, e.g., the molecular changes during substrate binding and enzymatic catalysis, how drug molecules interact with their target, and how modifications could alter the interactions or the mechanism. In general, structural studies of disease-related proteins play an important role in drug discovery, not only by providing fundamental information about protein function or specific drug binding, but also by presenting more information for hit-to-lead optimisation or for the development of chemoinformatics and medicinal chemistry programs [205,206]. In the search for, e.g., pharmacological chaperones, well-defined crystal structures are particularly important in docking-based in silico screenings to identify and characterize binders. When working with HMBS, an enzyme with a complex multi-step mechanism, we would benefit even more if structural and mechanistic details beyond ES_2_ intermediate state formation were known [75].

There are several published structures of HMBS (Table 1); however, until 2018, only structures in the holo-state had been published. The structure of HMBS in the ES_2_ intermediate state demonstrated how specific areas of the enzyme, as well as the growing pyrrole chain, moved during the elongation [75], adding to the complexity of the enzyme mechanism. In particular, the loop covering the active site (residues 58–74) is thought to be important in the catalytic process. This loop has been suggested by *in silico* analysis to move from a ‘closed’ to an ‘open’ conformation during the elongation process, in agreement with its relevant role in catalysis [207]. The structure of the loop cannot be defined in most of the crystal structures, probably because of its dynamic properties, and has only been detected in the ‘closed’ state in the crystal structure of *A. thaliana* HMBS (PDB 4HTG) [72], and more recently in the ‘open’ state of the structures of human wild-type (PDB 5M6R) and p.Arg173Trp mutant (PDB 7AAK) [75,76]. However, the function, and participation of this dynamic loop in the catalytic mechanism are still unclear.

To date, only two mutant structures of human HMBS have been solved. The p.Arg167Gln structure (PDB 3EQ1) [71] was crystallized in the holo-state, and the mutant residue was not well-defined in the electron density. Supporting experiments, however, showed that the Arg167Gln mutant hinders the turnover and product release [71]. The most recent discovery is the structure of the p.Arg173Trp AIP-associated mutant (PDB 7AAK) in the trapped ES_2_ catalytic intermediate state, which represents the first structure of a catalytically arrested elongation intermediate and pinpoints the importance of Arg173 in catalysis [76]. Compared to wild-type HMBS in the E state, the structure of this mutant also reveals large rearrangements of the loops around the active site.

Approaches aimed at the stabilisation of wild-type HMBS present in most AIP patients are being studied [133] (see above). Nonetheless, small-molecule-based therapies aimed at the activation and stabilisation of mutant forms also represent interesting options, with both a higher correction potential and the possibility of patient-tailored intervention. The conformational variability of HMBS, which explores several enzyme intermediates along the elongation process (E_(holo)_ and ES_1–4_; Figure 3), is clearly altered in p.Arg173Trp, concomitant with a defective elongation mechanism. This mutant may be a target for pharmacological chaperones that specifically bind to the ES_2_ conformer, hopefully shifting the equilibrium to a repopulated conformer ensemble, bypassing the elongation halt towards ES_3_ [132,183,185]. The effectiveness of high-resolution mass spectrometry to separate and identify the intermediates [76], and its adaptability to high-throughput screening, readily encourages the search for these kind of pharmacological chaperones for specific AIP mutants.

Further structural studies are needed even if the dynamic nature of the enzyme makes it challenging for X-ray crystallography. The use of complementary methods such as nuclear magnetic resonance (NMR) and cryo electron microscopy (cryo EM), combined with X-ray crystallography, might lead us closer to understanding the mechanism. Recently, NMR experiments with isotope-enriched HMBS have indeed revealed local changes in the protein structure between different reaction intermediates [75]. The recent methodological and technological advances in cryo EM, including the labelling and scaffolding of small proteins as well as single-particle cryo EM, push the size limit down and allow for higher flexibility in the proteins to be studied, increasing the chances of the structures of the catalytic states of HMBS being solved by this technique [208,209].

By choosing a comprehensive combination of structural and biophysical characterisation methods, much can still be learned from this system. Therefore, despite the challenges rising from the limitations of the structural methods and the nature of HMBS, we believe that mechanistic details of HMBS and AIP-associated mutants will become clear in the near future.

## 6. Conclusions

In this review, we have provided an overview of treatments currently in use in clinical practice and therapies under development or in the pipeline. There is a clear clinical need for new treatments, particularly treatments that may be used prophylactically to prevent the development of symptomatic disease and long-term complications in susceptible *HMBS* variant carriers. Gene- and mRNA-related therapies, presently under development, have a large potential to improve patient care. Novel therapies under investigation based on the stabilisation and/or activation of wild-type HMBS and mutants associated with AIP, such as pharmacological chaperones that may increase the half-live and functionality of the enzyme in vivo, also have the potential of prophylactic correction of the disease. Moreover, increased efforts to unravel the structure–function relationships and the catalytic mechanism of HMBS, as well as the kinetic effects of mutations, are also expected to facilitate the development of precision therapeutic interventions in AIP.

## Data Availability

Not applicable.

## Supplementary Materials

The following are available online at https://www.mdpi.com/1422-0067/22/2/675/s1.

## Abbreviations

AAV	adeno-associated virus
ALA	δ-aminolaevulinic acid
ALAD	δ-aminolaevulinic acid dehydratase
ALAS	δ-aminolaevulinic acid synthase
CYP	cytochrome P450-dependent oxidase
DPM	Dipyrromethane
E, E_(holo)_	holoenzyme
ES_1–4_	Enzyme-substrate intermediates, with 1 to 4 substrate units
GABA	γ-aminobutyric acid
GATs	GABA transporters
HMB	1-hydroxymethylbilane
HMBS	Hydroxymethylbilane synthase
PBG	Porphobilinogen
PEPT2	peptide transporter 2
PGC-1α	peroxisome proliferator-activated receptor γ coactivator 1α
ROS	reactive oxygen species
siRNA	small interference RNA
*T*_m_	half-denaturing temperature
UPS	ubiquitin-proteasome system
