# Supplementary material for: Acute Intermittent Porphyria: An Overview of Therapy Developments and Future Perspectives Focusing on Stabilisation of HMBS and Proteostasis Regulators
Source: Int J Mol Sci. 2021 Jan 12;22(2):675. doi: 10.3390/ijms22020675 (PMC7827610; doi:10.3390/ijms22020675)
Supplement: Supplementary file 1 [file ijms-22-00675-s001.pdf]

Supplemental Table S1. Summary of missense mutations with classification, outcome and the conclusion from original research article. For consistency, the localisation was mapped to human HMBS[1] for each entry. Variants highlighted in grey are predicted to have no pathogenicity.

Amino acid change	Classification			Outcome			Original conclusion	
	Catalytic effect	Unstable conformation	Both	Prokaryotic expression (% WT[2] activity)	Other experimental approaches	Localisation (on the human HMBS[1])		
p.Met1Ile	X			0%	TS[3]	Domain 1, loop, surface	—	[12]
p.Gly24Ser	X			7%	WB[4]	Domain 1, β sheet, buried	—	[13]
p.Arg26Cys	X			—	*In silico*[5]	Domain 1, β sheet, active site	Altered interaction with DPM	[144]
p.Arg26Cys	X			—	*In silico*	Domain 1, β sheet, active site	Altered interaction with reaction intermediate and DPM but away from the active site on 3D structure	[8]
p.Arg26Cys	X			0.3%	TS / *In silico* / SDS-PAGE	Domain 1, β sheet, active site	Altered interaction with DPM	[135]
p.Arg26Cys	X			0.3%	*In silico*	Domain 1, β sheet, active site	No cofactor assembly	[62]
p.Arg26Cys	X			5%	WB	Domain 1, β sheet, active site	—	[13]
p.Arg26His	X			0.3%	*In silico*	Domain 1, β sheet, active site	No cofactor assembly	[62]
p.Arg26His	X			0.2%	TS / *In silico* / SDS-PAGE	Domain 1, β sheet, active site	Altered interaction with DPM	[135]
p.Arg26His	X			3%	WB	Domain 1, β sheet, active site	No pyrrole chain elongation	[13]
p.Ser28Asn	X			0.8%	*In silico*	Domain 1, loop, surface	—	[62]
p.Ser28Asn	X			4%	WB	Domain 1, loop, surface	—	[13]
p.Leu30Pro	X			0.2%	TS / WB / *In silico*	Domain 1, α helix, surface, at the edge of active site	—	[139]
p.Leu30Phe	X			3%	WB	Domain 1, α helix, surface, at the edge of active site	—	[13]
p.Ala31Thr	X			Data not shown	—	Domain 1, α helix, buried	—	[143]
p.Ala31Pro	X			0.6%	*In silico*	Domain 1, α helix, buried	Altered elongation mechanism	[62]
p.Arg32Pro	X			<1%	TS / *In silico*	Domain 1, α helix, surface, at the edge of active site	Distortion of active site	[136]
p.Gln34Arg	X			0.7%	*In silico*	Domain 1, α helix, active site	No pyrrole chain elongation	[62]
p.Gln34Lys		X			*In silico*	Domain 1, α helix, active site	Secondary structure alteration	[145]
p.Gln34Lys	X			0.2%	*In silico*	Domain 1, α helix, active site	No pyrrole chain elongation	[62]
p.Gln34Pro	X			1.3%	*In silico*	Domain 1, α helix, active site	Secondary structure alteration	[140]
p.Thr35Met	X			3.8%		Domain 1, α helix, buried		[141]

p.Thr35Met				—	*In silico*	Domain 1, α helix, buried	Far from active site and no interaction with reaction intermediate and DPM	[8]
p.Thr35Met	X			11%	WB	Domain 1, α helix, buried	—	[13]
p.Thr59Ile				77%; (TS 51±16%)	TS	Domain 1, α helix, surface	No defect in enzyme kinetic or stability; Predicted pathogenicity: Benign	[12]
p.Thr59Ile		X		81%; (TS 10%)	TS / *In silico*	Domain 1, α helix, surface	—	[136]
p.Phe77Leu	X			0.1%	TS / WB / *In silico*	Domain 1, active site loop, surface	—	[139]
p.Thr78Pro			X	2.5%	*In silico*	Domain 1, α helix, surface	—	[148]
p.Glu80Gly			X	2.4%	*In silico*	Domain 1, α helix, surface	—	[148]
p.Glu86Val				94%; (TS 45±15%)	TS	Domain 1, α helix, surface	No defect in enzyme kinetic or stability; Predicted pathogenicity: Benign	[12]
p.Glu86Val				93%	WB	Domain 1, α helix, surface	—	[13]
p.Val93Phe		X		1.1%	TS / *In silico*	Domain 1, β sheet, buried	Secondary structure alteration	[134]
p.Ser96Phe	X			1.0%	*In silico*	Domain 1, loop, active site	No pyrrole chain elongation	[62]
p.Ser96Phe	X			3%	WB	Domain 1, loop, active site	—	[13]
p.Lys98Asn	X			<1%	—	Domain 1, loop, active site	Altered interaction with DPM	[146]
p.Lys98Arg	X			0.7%	*In silico*	Domain 1, loop, active site	No cofactor assembly	[62]
p.Asp99Asn	X			—	*In silico*	Domain 1, loop, active site	Altered interaction with reaction intermediate and DPM but away from the active site on 3D structure	[8]
p.Asp99Gly	X			3.2%	*In silico*	Domain 1, loop, active site	No cofactor assembly	[62]
p.Asp99His	X			3.3%	*In silico*	Domain 1, loop, active site	No cofactor assembly	[62]
p.Gly111Arg				—	*In silico*	Domain 1, β sheet, buried	Far from active site and no interaction with reaction intermediate and DPM	[8]
p.Gly111Arg	X			4%	WB	Domain 1, β sheet, buried	—	[13]
p.Ala112Pro			X	0.1%	TS / WB / *In silico*	Domain 1, β sheet, buried	—	[139]
p.Arg116Trp			X	1.4%	TS / *In silico*	Loop between domain 1 and 2, side chain buried	—	[134]
p.Arg116Trp			X	—	*In silico*	Loop between domain 1 and 2, side chain buried	Altered interaction with DPM	[149]

p.Arg116Trp		X	0.5%	TS / *In silico* / SDS-PAGE	Loop between domain 1 and 2, side chain buried	Strong conformational defect and incapacity of association with DPM	[37]
p.Arg116Trp		X	—	*In silico*	Loop between domain 1 and 2, side chain buried	Strong conformational defect and incapacity of association with DPM	[8]
p.Arg116Trp	X		4%	WB	Loop between domain 1 and 2, side chain buried	—	[13]
p.Ala122Asp		X	0.2%	TS / WB / *In silico*	Domain 2, β sheet, buried	—	[139]
p.Ala122Asp		X	—	*In silico*	Domain 2, β sheet, buried	Altered interaction with reaction intermediate and DPM	[8]
p.Val124Asp	X		4%	WB	Domain 2, β sheet, buried	—	[13]
p.Lys132Asn			97%	TS / *In silico* / SDS-PAGE	Domain 2, loop, surface	No defect in enzyme kinetic, conformation or stability	[37]
p.Thr145Ile	X		0.4%	TS / WB / *In silico*	Domain 2, β sheet, partially buried, active site	—	[139]
p.Thr145Ile	X		—	*In silico*	Domain 2, β sheet, partially buried, active site	Altered interaction with reaction intermediate and DPM	[8]
p.Ser147Pro	X		0.2%	*In silico*	Domain 2, loop, active site	—	[62]
p.Arg149Leu	X		0.1%	*In silico*	Domain 2, α helix, active site	No cofactor assembly	[62]
p.Arg149Gln	X		0.5%	*In silico*	Domain 2, α helix, active site	No cofactor assembly	[62]
p.Arg149Leu	X		—	*In silico*	Domain 2, α helix, active site	Altered interaction with DPM	[143]
p.Arg149Leu	X		5%	WB	Domain 2, α helix, active site	—	[13]
p.Arg149Gln	X		5%	WB	Domain 2, α helix, active site	—	[13]
p.Leu154Pro		X	0.1%	TS / WB / *In silico*	Domain 2, α helix, buried	—	[139]
p.Arg167Gln		X	0.7%	TS / *In silico*	Domain 2, loop, surface	—	[142]
p.Arg167Gln	X		1%	TS	Domain 2, loop, surface	—	[12]
p.Arg167Gln	X		1%	*In silico*	Domain 2, loop, surface	No structural changes<br>Product release blocked	[62]
p.Arg167Gln		X	—	*In silico*	Domain 2, loop, surface	Alteration of conserved amino acid residue	[145]
p.Arg167Trp	X		—	*In silico*	Domain 2, loop, surface	Altered interaction with DPM	[144]
p.Arg167Trp	X		4.2%	TS / *In silico* / SDS-PAGE	Domain 2, loop, surface	Altered elongation mechanism	[37]
p.Arg167Trp	X		—	*In silico*	Domain 2, loop, surface	Altered interaction with reaction intermediate and DPM	[8]

p.Arg167Trp	X			3%	TS	Domain 2, loop, surface	—	[12]
p.Arg167Trp	X			2.3%	*In silico*	Domain 2, loop, surface	No structural changes. Product release blocked	[62]
p.Gly168Trp	X			—	*In silico*	Domain 2, loop, surface	Altered interaction with reaction intermediate and DPM but away from the active site on 3D structure	[8]
p.Leu170Pro	X			<0.1%	TS / WB / *In silico*	Domain 2, α helix, surface, at the edge of active site	—	[139]
p.Arg173Gln	X			0.6%	TS / *In silico*	Domain 2, α helix, active site	—	[142]
p.Arg173Gln	X			—	*In silico*	Domain 2, α helix, active site	Altered interaction with DPM	[144]
p.Arg173Gln				0.15%	TS / *In silico* / SDS-PAGE	Domain 2, α helix, active site	Altered interaction with DPM	[135]
p.Arg173Gln	X			0.4%	*In silico*	Domain 2, α helix, active site	No cofactor assembly	[62]
p.Arg173Trp	X			—	*In silico*	Domain 2, α helix, active site	Altered interaction with DPM	[149]
p.Arg173Trp			X	0.6%	TS / *In silico* / SDS-PAGE	Domain 2, α helix, active site	Altered interaction with reaction intermediate and altered elongation	[37]
p.Arg173Trp	X			0.7%	*In silico*	Domain 2, α helix, active site	—	[62]
p.Arg173Trp			X	—	*In silico*	Domain 2, α helix, active site	Altered interaction with reaction intermediate and DPM	[8]
p.Leu177Arg	X			—	*In silico*	Domain 2, α helix, buried	Alteration of conserved amino acid residue	[145]
p.Asp178Asn		X		81% (TS 70%)	TS / *In silico*	Domain 2, α helix, surface	—	[136]
p.Arg195Cys	X			3%	TS	Domain 2, α helix, active site	—	[12]
p.Arg195Cys	X			3%	*In silico*	Domain 2, α helix, active site	No cofactor assembly	[62]
p.Arg195Cys	X			0%	WB	Domain 2, α helix, active site		[13]
p.Arg201Trp		X		41.6% Thermolabile	TS / *In silico*	Domain 2, α helix, surface	—	[134]
p.Gln204Lys		X		46% Thermolabile	TS / *In silico* / SDS-PAGE	Domain 2, β sheet, surface	—	[135]
p.Met212Val	X			1.7%	*In silico*	Domain 2, loop, surface	Loss of mobility and block of substrate entrance	[149]
p.Val215Glu		X		30%	TS / *In silico* / SDS-PAGE	Loop between domain 1 and 2, buried, close to cofactor binding loop	Altered elongation mechanism	[37]
p.Val215Met	X			19%	TS / *In silico*	Loop between domain 1 and 2, buried, close to cofactor binding loop		[136]
p.Gln217His	X			—	*In silico*	Loop between domain 1 and 2, active site	Altered interaction with reaction intermediate and DPM	[8]

p.Gln217Arg	X			—	*In silico*	Loop between domain 1 and 2, active site	Alteration of active-site residues Lys98 and Arg150 orientation	[62]
p.Gln217His	X			—	*In silico*	Loop between domain 1 and 2, active site	Alteration of active-site residues Lys98 and Arg150 orientation	[62]
p.Gln217His	X			5%	WB	Loop between domain 1 and 2, active site		[13]
p.Gly218Arg	X			—	*In silico*	Loop between domain 1 and 2, buried	Altered interaction with reaction intermediate and DPM	[8]
p.Gly218Arg	X			0.1%	*In silico*	Loop between domain 1 and 2, buried		[62]
p.Ala219Pro	X			—	*In silico*	Loop between domain 1 and 2, buried	Altered interaction with reaction intermediate and DPM	[8]
p.Gly221Asp	X			—	*In silico*	Domain 1, loop, buried	Altered interaction with reaction intermediate and DPM but away from the active site on 3D structure	[8]
p.Val222Met	X			—	*In silico*	Domain 1, β sheet, buried	Disruption of interaction in the active site	[147]
p.Val224Glu			X	0.9%	TS / WB / *In silico*	Domain 1, β sheet, buried		[139]
p.Arg225Gln				102% (TS 31±10%)	TS	Domain 1, β sheet, surface	No defect in enzyme kinetic or stability; Predicted pathogenicity: Benign	[12]
p.Asp230Tyr				88% (TS 50±16)	TS	Domain 1, α helix, surface	No defect in enzyme kinetic or stability; Predicted pathogenicity: Benign	[12]
p.Val235Glu	X			3%	WB	Domain 1, α helix, buried		[13]
p.Leu238Pro				—	*In silico*	Domain 1, α helix, surface	Far from active site and no interaction with reaction intermediate and DPM	[8]
p.Asp240Gly			X	5%	TS / WB / *In silico*	Loop between domain1 and 3, surface	—	[139]
p.Cys247Arg	X			2%	WB	Domain 3, α helix, buried	—	[13]
p.Cys247Phe				11 %	TS / *In silico*	Domain 3, α helix, buried	—	[134]
p.Glu250Asp			X	0.5%	TS / *In silico* / SDS-PAGE	Domain 3, α helix, buried	—	[135]
p.Glu250Lys		X		—	*In silico*	Domain 3, α helix, buried	—	[149]
p.Glu250Val	X			4%	WB	Domain 3, α helix, buried	—	[13]
p.Glu250Gln	X			2%	WB	Domain 3, α helix, buried	—	[13]
p.Glu250Ala	X			4%	WB	Domain 3, α helix, buried	—	[13]
p.Ala252Val		X		61% Thermolabile	TS	Domain 3, α helix, buried	—	[12]
p.His256Asn	X			—	*In silico*	Domain 3, loop, surface	Alteration of conserved amino acid residue	[143]

p.His256Tyr	X		5%	WB	Domain 3, loop, surface	—	[13]
p.Gly260Asp	X		—	*In silico*	Domain 3, cofactor binding loop	Impairment of chain elongation	[62]
p.Val267Met	X		4%	WB	Domain 3, β sheet, buried	—	[12]
p.Leu278Pro		X	—	*In silico*	Domain 3, loop, buried	Folding defect	[147]
p.Arg321His			122%<br>(TS 31±10%)	TS	Domain 3, loop, buried	No defect in enzyme kinetic or stability;<br>Predicted pathogenicity: Benign	[12]
p.Arg321His			96%	WB		—	[13]
p.Ala330Pro		X	—	*In silico*	Domain 3, α helix, surface	Alteration in secondary structure of the protein	[8]
p.Ala331Val		X	62%<br>Thermolabile	TS	Domain 3, α helix, buried	—	[12]
p.Gly335Ser	X		2.5%	*In silico*	Domain 3, α helix, buried	—	[140]
p.Ala347Pro		X	51%<br>Thermolabile	TS / WB /<br>*In silico*	Domain 3, α helix, buried	—	[139]
p.Asp359Asn			86%<br>(TS 42±13%)	TS	Domain 3, loop, surface	No defect in enzyme kinetic or stability;<br>Predicted pathogenicity: Benign	[12]
p.Asp359Asn			93%	WB	Domain 3, loop, surface	—	[13]

[1]Localisation is based on human HMBS crystal structure 7AAJ [76]
[2]WT, wild-type
[3]TS, Thermostability assays
[4]WB, Western blot
[5]*In silico*, mapping of the mutation on the human HMBS crystal structure and bioinformatical prediction
